# The Virus–Host Interplay in Junín Mammarenavirus Infection

**DOI:** 10.3390/v14061134

**Published:** 2022-05-24

**Authors:** Giovanna Lucrecia Gallo, Nora López, María Eugenia Loureiro

**Affiliations:** Centro de Virología Humana y Animal (CEVHAN), Consejo Nacional de Investigaciones Científicas y Técnicas (CONICET), Universidad Abierta Interamericana (UAI), Buenos Aires C1287, Argentina; giovannalgallo@gmail.com (G.L.G.); NoraMabel.Lopez.Cevhan@UAI.edu.ar (N.L.)

**Keywords:** Junín virus, Argentine hemorrhagic fever, arenavirus, host–virus interactions, entry, replication, assembly, budding, immune response

## Abstract

Junín virus (JUNV) belongs to the *Arenaviridae* family and is the causative agent of Argentine hemorrhagic fever (AHF), a severe human disease endemic to agricultural areas in Argentina. At this moment, there are no effective antiviral therapeutics to battle pathogenic arenaviruses. Cumulative reports from recent years have widely provided information on cellular factors playing key roles during JUNV infection. In this review, we summarize research on host molecular determinants that intervene in the different stages of the viral life cycle: viral entry, replication, assembly and budding. Alongside, we describe JUNV tight interplay with the innate immune system. We also review the development of different reverse genetics systems and their use as tools to study JUNV biology and its close teamwork with the host. Elucidating relevant interactions of the virus with the host cell machinery is highly necessary to better understand the mechanistic basis beyond virus multiplication, disease pathogenesis and viral subversion of the immune response. Altogether, this knowledge becomes essential for identifying potential targets for the rational design of novel antiviral treatments to combat JUNV as well as other pathogenic arenaviruses.

## 1. Introduction

Junín virus (JUNV) is the causative agent of Argentine hemorrhagic fever (AHF), a severe hemorrhagic disease endemic to the fertile farming areas of Argentina [1]. JUNV is a rodent-borne virus, and *Calomys musculinus* has been identified as its principal reservoir. Humans become infected through direct contact with or by inhalation of aerosolized rodent body fluids or excreta, mostly during agricultural work in harvest season. The emergence of AHF in Argentina in the 1950s is believed to be the consequence of favorable conditions for the unleashed propagation of *Calomys musculinus*. Since then, outbreaks with hundreds of cases have been reported every year; however, with the availability of the effective live-attenuated Candid#1 vaccine since the 1990s, the incidence of AHF has significantly decreased [2].

AHF disease initially starts with general flu-like symptoms, such as fever, malaise and headache. The virus replicates at the initial site of infection, generally the lungs, and further disseminates to other parenchymal tissues [3]. Hemorrhagic and neurologic complications develop, in severe cases, with mortality rates that can rise up to 20% in hospitalized patients. Treatment with immune plasma from convalescent patients, in standardized doses of neutralizing antibodies, is currently the antiviral therapy being used for AHF. This treatment has been demonstrated to drop case-fatality rates to 1% [4]. However, this strategy lacks efficacy if administered after 8 days of the onset of symptoms [5,6], and it is associated with a late neurological syndrome in approximately 10% of the treated AHF survivors [4,7]. The off-label FDA-approved nucleoside analog, ribavirin, which inhibits the viral polymerase, has also been evaluated in patients as an antiviral drug to combat JUNV infection; however, its efficacy is errant, and it has been shown to cause serious side effects [8,9].

Over the last years, numerous studies have oriented efforts toward searching for antiviral treatments against pathogenic mammarenaviruses [10]. As for JUNV, novel therapeutic targets have recently emerged from host- or virus-directed small-interfering RNA (siRNAs) and drug high-throughput screens (HTS) [11,12,13]. Several antiviral compounds showed promise in inhibiting JUNV infection either in vitro [13,14,15,16,17,18] or in animal models [19,20,21]. Most of them interfere in different stages of the JUNV cycle, ranging from virus entry [13,19] to viral genome replication [15,18,20] and egress [17]. Moreover, cocktails of available approved antiviral drugs have also been considered as a potential therapeutic approach. These antiviral cocktails stand as a promissory strategy to limit JUNV viral multiplication, as they could synergistically target different steps of the viral life cycle and lower the potential risk of the emergence of drug resistance [22]. Several HTS resulted in the identification of candidate compounds that not only display a potent antiviral activity against JUNV but also against a broad panel of mammarenaviruses [13,23,24,25]. In this sense, the development of recombinant pseudo-type platforms has allowed for the successful screening of libraries for broad-spectrum arenavirus entry inhibitors [13,24,26,27]. Likewise, the design of pan-arenavirus inhibitors, such as peptide-conjugated phosphorodiamidate morpholino oligomers (PPMOs), directed to target conserved regions within mammarenavirus genome RNA, showed to be effective against multiple members of the *Arenaviridae* family including JUNV [28]. Finally, recent investigations have also made progress in the development of antibodies for neutralizing therapies, either directly targeting JUNV [29,30,31,32,33] or preventing virus entry by occluding JUNV’s binding site on the cell [34,35,36]. Altogether, despite all these novel findings and promising approaches, an efficient alternative antiviral therapy is still vacant and the advancement in novel strategies for the treatment of AHF remains compelling.

JUNV belongs to the *Arenaviridae* family. These are enveloped viruses that replicate in the cytoplasm, with a negative-sense RNA genome consisting of two single-stranded segments named S (for Small, approximately 3.4 kb) and L (for Large, approximately 7.2 kb). Each segment encodes two proteins with opposite (ambisense) orientations. The S segment encodes the nucleoprotein (NP) and the glycoprotein precursor (GPC). GPC is cleaved by cellular proteases, rendering three subunits (GP1, GP2 and a stable signal peptide (SSP)) that remain associated and conform the mature envelope glycoprotein complex (GP) [37,38]. The L segment encodes the viral RNA-dependent RNA polymerase (L) and the matrix protein (Z) [39]. In each segment, the open reading frames, in opposite orientations, are separated by a noncoding intergenic region predicted to fold into strong stem-loop structures [40].

Arenaviruses are currently classified into four genera and infect mammals (mammarenaviruses), fish (antennaviruses) and snakes (hartmaniviruses and reptarenaviruses). Members of the *Mammarenavirus* genus are divided into old world (OW) and new world (NW) groups according to geographical distribution, antigenic properties and phylogenetic relationships [41]. OW mammarenaviruses include the prototypic lymphocytic choriomeningitis virus (LCMV), of worldwide distribution, as well as viruses causing hemorrhagic disease in humans such as Lassa virus (LASV), which is endemic in West Africa. The NW group includes highly pathogenic members causing human hemorrhagic fevers in South America, namely, JUNV, Machupo (MACV), Chapare, Guanarito and Sabia, along with nonpathogenic viruses such as Tacaribe virus (TCRV), a prototypic model of the NW group.

According to the World Health Organization (WHO), arenaviral HFs are listed as emerging diseases for which immediate research and identification of clinical targets are required [42]. Given the lack of FDA-approved vaccines and antiviral therapies to combat pathogenic mammarenavirus infections, a better understanding of the mechanisms underlying viral multiplication and pathogenesis has become imperative. In the recent years, significant reports accumulated regarding host factors that actively participate in different steps of the JUNV life cycle, as well as those being hijacked by the virus to subvert the host immune response. Therefore, here, we review current knowledge on virus–host interactions relevant for JUNV infection, which may contribute to future studies oriented to the rational design of novel therapeutic approaches.

## 2. Host–Virus Interactions Involved in JUNV Entry to the Cell

The JUNV cycle starts with the attachment of the viral particle to a host receptor on the cell surface. Thereafter, virus internalization followed by fusion between the viral and cellular membranes enables the release of the viral nucleocapsid into the cytoplasm and the initiation of the replication process. The tripartite JUNV’s GP is the viral entry key to the cell. The GP1 subunit recognizes and binds to the host receptor, whereas GP2 drives the fusion of viral and cellular membranes [37,43]. The SSP is essential for intracellular trafficking and proteolytic maturation of the GPC precursor and has a key role in membrane fusion [44,45].

### 2.1. Transferrin Receptor 1 Is the Preferred Receptor for JUNV Entry

**Figure 1:**
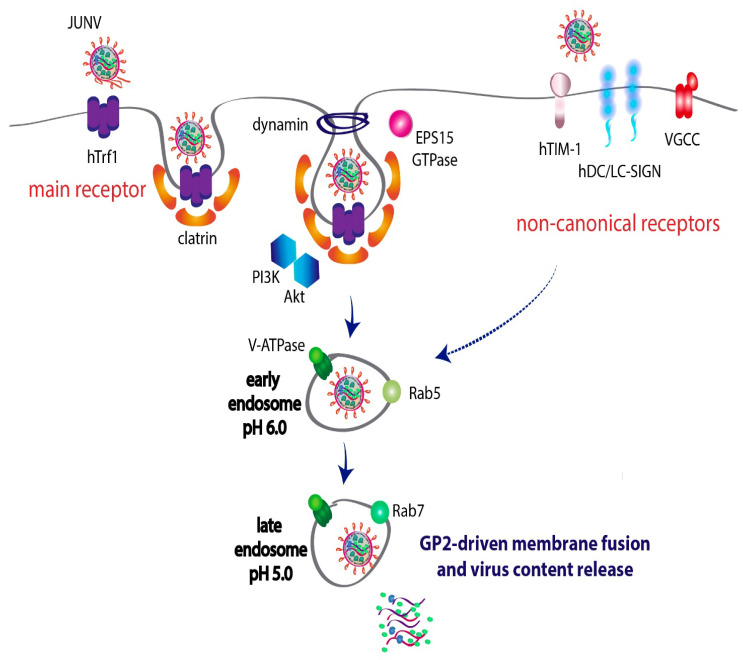
JUNV entry to the cell. JUNV uses hTrf1 (human Transferrin receptor 1) as its main receptor to enter the cell, but, under certain constraints, it utilizes alternative ports of entry. As noncanonical receptors, JUNV uses: (i) hTIM-1 phosphatidylserine receptor; (ii) lectin receptors, such as hDC-SIGN and hL-SIGN; (iii) voltage-gated calcium channels (VGCCs). Thereafter, JUNV is internalized through a clathrin-mediated endocytic pathway, which is dependent on dynamin II and EPS15 proteins, and the PI3K/Akt signaling pathway is early activated. Then, JUNV travels through the cellular endocytic pathway, specifically from Rab5-early (pH = 6.2–6.5) to Rab7-late (pH = 5.0–6.0) endosomes. Endosomal acidification is a necessary event to fulfill virus internalization and vacuolar-proton ATPases (V-ATPases), which acidify endosomes by pumping protons across membranes, strongly contributing to this process. Low pH triggers conformational changes in the arenavirus glycoprotein (GP), resulting in the exposure of a specific motif within the GP2 subunit that mediates fusion of the virion with host cell membranes. This enables the release of the viral content into the cytoplasm. TIM-1: T-cell immunoglobulin and mucin-domain 1; hDC-SIGN: human dendritic cell-specific intercellular adhesion molecule-3 grabbing nonintegrin; hL-SIGN: liver/lymph node-specific ICAM-3-grabbing nonintegrin; EPS15: epidermal growth factor receptor substrate 15.

JUNV uses human transferrin receptor 1 (hTfR1) as its main cellular receptor [46] (Figure 1). Similarly, other pathogenic NW mammarenaviruses, such as MACV, the causative agent of Bolivian hemorrhagic fever, use hTfR1 as its primary entry receptor in human cells [46]. In contrast, the nonpathogenic TCRV binds TfR1 orthologues of several mammalian species but not the human TfR1; despite this, TCRV is still capable of infecting human cells independently of the presence of hTfR1 [47]. Guinea pigs and nonhuman primates experimentally infected with JUNV have shown to better reproduce the disease observed in humans [48,49]. Recent evidence demonstrated that guinea pig TfR1 (gpTfR1) is the preferred animal receptor and that hamster and mouse orthologues fail to support viral entry [50]. Notably, both the pathogenic and nonpathogenic NW mammarenaviruses are capable of infecting mice as well as *Mus musculus* primary cultures and cell lines, although they are unable to bind mouse TfR1 (mTfR1) [50,51,52]. This envisions the idea that binding of the viral GP to a specific TfR1 orthologue may contribute to defining whether a species is susceptible to severe disease or may just serve as a reservoir. Notably, mutation of one residue within hTfR1 makes it a functional receptor for TCRV, opening the intriguing question of whether nonpathogenic arenaviruses may emerge as possible human pathogens [47].

### 2.2. JUNV Alternatively Uses Noncanonical Receptors to Enter the Cell

Cell surface receptors such as phosphatidylserine (PS) receptors, C-type lectins and voltage-gated calcium channels (VGCCs), have been implicated as alternative sites of entry during JUNV infection [11,53,54] (Figure 1). First, many enveloped viruses utilize PS receptors to enter into the endosomal compartments, a mechanism known as “apoptotic mimicry”. Experiments applying a platform of pseudo-typed retroviruses and virus-like particles (VLPs) with different viral envelope glycoproteins, ranging from NW mammarenaviruses to filovirus, flavivirus and alphavirus, demonstrated that JUNV uses the human T-cell immunoglobulin and mucin-domain (hTIM-1) PS receptor for cell entry [53]. TIM receptors are cell surface glycoproteins that display an extracellular immunoglobulin variable-like domain (IgV), bearing a structural pocket with high affinity for PS [55]. Evidence showed that hTIM-1 overexpression increased JUNV-GP pseudo-virus infection in human 293T cells compared to the respective control [53]. In contrast, LASV and LCMV OW arenaviruses, which use alpha-dystroglycan (α-DG) as their main cellular receptor [56], only weakly utilized hTIM-1 [53]. In this regard, it has been proposed that hTIM-1 mostly acts as an accessory attachment factor by associating with PS on the virions surface and that its role may be influenced by the virus accessibility to its respective primary receptor [53], although TIM-1 could still serve as a LASV receptor in the absence of α-DG or under conditions where it is inadequately glycosylated [57]. Interestingly, this phenomenon would not be limited to the *Arenaviridae* family, as a recent report for severe acute respiratory syndrome coronavirus 2 (SARS-CoV-2) suggests that PS receptors enhance infection when low levels of the main receptor, the angiotensin-converting enzyme 2 (ACE-2), are expressed [58].

Experiments using JUNV, as well as pseudo-typed retroviral particles carrying functional JUNV GP, demonstrated that viral infection is also enhanced by the presence of human dendritic cell-specific intercellular adhesion molecule-3 grabbing nonintegrin (hDC-SIGN) or its homologue liver/lymph node-specific ICAM-3-grabbing nonintegrin (DC-SIGN or L-SIGN). This enhancement was independent of the presence of hTfR1 [54]. Both hDC-SIGN and hL-SIGN are cell surface transmembrane proteins belonging to the calcium-dependent (C-type) family of lectins, which bind to multiple viruses through high-mannose *N*-glycans [59,60]. Though relatively short, the cytoplasmic tail of the lectin carries several motifs involved in signaling, endocytic internalization and intracellular trafficking [59]. It is believed that viruses may replace such biological functions to trigger different cell signaling cascades for their own sorting into a specific endocytic pathway. As for JUNV, hDC-SIGN and hL-SIGN were able to solely sustain viral infection independently of the presence of hTfR1, but, nevertheless, none of these lectin receptors showed higher efficiency than hTfR1 [54].

VGCCs are hetero-multimeric protein channels that convert membrane electrical signals to intracellular transient calcium waves. They build up from the assembly of the pore-forming α1 subunit and the regulatory α2δ, β and γ subunits [61]. It has been reported that VGCCs, or their subunits, facilitate efficient JUNV and TCRV entry into mouse cells [62], presumably by promoting virus–cell membrane fusion [11]. Moreover, there is evidence demonstrating the physical interaction between GPs of other pathogenic arenaviruses (MACV, LCMV and LASV) and α1s and α2δ2 subunits of VGCCs. This suggests that JUNV’s GP may probably also associate to both subunits on the cell surface to mediate virus entry. In line with this, evidence concurs that α1s haplo-insufficiency turns both mouse cells and mice more resistant to infection by JUNV, reinforcing the important role of VGCCs in viral entry [62].

### 2.3. JUNV Utilizes the Clathrin-Mediated Endocytic Pathway to Fulfill Viral Entry

Upon specific interaction with the respective cell receptor, most viruses can be internalized into the host cell through alternative endocytic pathways such as (i) clathrin-coated pits, (ii) caveolae, (iii) lipid rafts membrane microdomains or (iv) macropynocitosis [63,64]. In the case of JUNV, it has been well characterized that viral entry is dependent on clathrin-mediated endocytosis [65]. Electron microscopy studies showed JUNV particles localizing within invaginations of the plasma membrane and vesicles resembling clathrin-coated pits. Moreover, compounds impairing clathrin-driven endocytosis reduced virus internalization without affecting virion binding, whereas those inhibiting caveola-mediated endocytosis were unable to block viral entry [65]. Besides, further studies confirmed that JUNV entry into host cells is dependent on dynamin II and on epidermal growth factor receptor substrate 15 (EPS15), two factors that are highly associated with the classical clathrin-coated endocytic pathway [66,67]. Of note, it was also demonstrated that the integrity of the actin cytoskeleton, as well as the flexibility of a dynamic microtubule network, are jointly required to sustain the progression of JUNV entry in early infection [68]. These findings are in agreement with evidence showing that an adequate assembly of the actin microfilaments directly impacts on different stages of the clathrin-coated vesicle formation, including coated pit genesis, constriction and internalization [69]. Altogether, these observations support clathrin-mediated endocytosis as the mechanism opted by JUNV to enter the cell.

### 2.4. JUNV Travels through Rab5-Early and Rab7-Late Endosomes before Membrane Fusion

JUNV travels within the cell through a progressive movement along the endocytic pathway. Early reports pointed out the requirement of endosomal acidification as a necessary event to fulfill virus internalization. Indeed, cell–cell fusion assays demonstrated that a pH of approximately 5 is the optimum to mediate fusion of the JUNV envelope with the cellular membrane [70]. Treatment with bafilomycin A1 during the first two hours post-infection, which targets vacuolar-proton ATPases (V-ATPases) that acidify endosomes by pumping protons across membranes [71], significantly decreased virus yields by preventing virus penetration [72]. In addition, JUNV was shown to use both Rab5-mediated early endosomes and Rab7-mediated late endosomes during the infection process [66]. Rab GTPases are organelle-specific markers that drive the maturation of endosomes by recruiting proteins involved in trafficking within the cytoplasm [73]. Cumulative evidence indicates that low pH triggers conformational changes in the arenavirus GP2, resulting in the exposure of a specific motif that mediates fusion of the virion with host cell membranes [74,75,76,77]. Considering that the pH within early and late endosomes range between 6.2–6.5 and 5.0–6.0, respectively, it is therefore conceivable that JUNV requires a Rab5-to-Rab7 endosome transition to direct the virus to a late endosomal compartment. This, in turn, allows an optimal environment for membrane fusion to accomplish virus penetration into the cell.

### 2.5. TRIM-2 Protein Restricts JUNV Entry into the Cell

The tripartite-motif (TRIM) family of proteins is characterized by the presence of a highly conserved tripartite motif at the N-termini. This motif is composed of a RING domain with ubiquitin ligase activity, one (or two) zinc-binding B-boxes and a coiled-coil domain, involved in protein–protein interactions. In humans, this family includes more than 70 members, many of which operate as antiviral restriction factors. TRIMs have been described as both constitutively present or interferon (IFN)-inducible antiviral molecules, and they either tackle different steps of the virus cycle, such as entry, transcription and budding, or they intervene in the innate immune response [78,79,80]. TRIM-2 knockdown experiments using JUNV or pseudo-typed retroviruses bearing JUNV’s GP on their surface revealed that TRIM-2 restricts viral infection at the entry stage, more specifically, at a post-binding step [81]. Moreover, experiments using TRIM-2-knockout mice confirmed that TRIM-2 also suppresses viral infection in vivo. In addition, the signal regulatory protein α (SIRPA), one of the TRIM-2 interacting partners, known as a modulator of phagocytosis, also decreased JUNV infection at the internalization step, as knockout of SIRPA resulted in primary mouse cells and mice to be more susceptible to JUNV infection [81,82]. Overall, the role of TRIM-2 and its partner SIRPA in limiting viral internalization has arisen as a novel antiviral function of these host factors.

## 3. JUNV Genome Replication, Assembly and Budding

The Mammarenaviruses viral genome is enwrapped by multiple copies of the NP, forming viral ribonucleoprotein complexes (vRNP), also known as nucleocapsids. These nucleocapsids tightly associate to the L RNA-dependent RNA polymerase, assembling into flexible higher-order multimeric structures [83] and constituting the biologically active units for transcription of subgenomic viral messenger RNA (mRNA) and for viral genome replication [84,85,86]. NP and L proteins are the minimum viral factors required for the onset of the replication process and are the first proteins synthesized. On the other hand, GPC and Z proteins comprise the late gene products in the viral cycle [39,84,85]. Z stands as a modulator of viral genome replication and transcription by directly binding the L polymerase and thereby inhibiting its activity [87,88,89,90,91]. In addition, Z matrix protein is considered a key player in the mammarenavirus morphogenesis process. The packaging of RNPs into infectious viral particles is driven by Z, which binds NP and recruits nucleocapsids to budding sites [92,93]. Importantly, the integrity of the central RING domain and the C-terminal domain of Z are required to sustain its association with NP [93], while the N-terminal domain of Z works as an anchor to the plasma membrane [90]. Thereafter, the combined interactions between Z–NP complexes and GP are thought to be necessary to fulfill efficient viral assembly, enabling a correct incorporation of GP into infectious particles [93,94]. In accordance with previous reports in OW arenaviruses [95,96], JUNV Z also has the capacity to self-assemble and is able to induce the formation of viral particles in the absence of other viral proteins [93,97]. Therefore, Z protein is considered the main driving force in the viral budding process.

### 3.1. JUNV Replicates in Discrete Cytosolic Structures

To accomplish viral replication, all viral components spread in the cytosol need to relocate to definite replicative sites that offer the adequate conditions required by the virus and guarantee physical and functional separation from the cell’s own physiological processes. There is evidence that JUNV replicates in discrete puncta structures within the cytoplasm called replication–transcription complexes (RTCs), where newly synthesized viral RNAs and NP localize [98]. In TCRV-infected cells, these structures were also shown to contain several host ribosomal proteins as well as the eukaryotic initiation factors (eIFs) eIF4G and eIF4A. Their association with phosphatidylinositol-4-phosphate (PI4P), their sensitivity to nonionic detergents and their buoyant density suggest that these viral RTCs might require rearrangements of cellular membranes for their assembly [98]. Similarly, it was demonstrated for the OW LCMV that the S segment genomic and antigenomic RNAs, along with NP, colocalize in highly dynamic subcellular distinct structures with small foci, which latercoalesce into larger perinuclear foci [99]. In this regard, it is conceivable that JUNV RTCs might correlate with other negative-stranded RNA viral factories shown to be liquid-like cytoplasmic organelles originated through biomolecular condensation to increase the efficiency of viral RNA synthesis [100,101,102,103]. However, further investigation is required to fully characterize the nature of these structures.

Interestingly, TCRV RTCs were also reported to colocalize with the stress granule (SG)-associated Ras GTPase-activating protein-binding protein 1 (G3BP1) [98], a phosphorylation-dependent endoribonuclease that modulates RNA metabolism and is recruited to SGs in cellular hostile conditions [104]. Likewise, in JUNV-infected cells, cytoplasmic G3BP1 was found to be concentrated in punctuated structures colocalizing with NP [105], and NP-G3BP1 interaction was validated by coimmunoprecipitation experiments, presumably suggesting that G3BP1 could be recruited to the replication and transcription factories of JUNV [105]. SG formation is triggered by the phosphorylation of eIF2α factor, which causes a shutdown of protein translation in response to cell damage or stress [106]. In reference to this, it was demonstrated that JUNV is capable of impairing SG formation by inhibiting eIF2α phosphorylation upon cell treatment with arsenite [107], which induces the production of reactive oxygen species (ROS) that cause cell damage. However, whether JUNV modulates SG formation by recruiting G3BP1 to RTCs and whether G3BP1 participates in viral RNA replication still needs to be clarified.

### 3.2. Role of DEAD-Box RNA Helicase 3 (DDX3) in JUNV Replication

The multifunctional roles of NP and Z in different stages of the viral cycle [108,109] make these two viral proteins an excellent starting point to study JUNV–host interactions. Recent large-scale proteomic approaches have identified novel human protein targets that interact with JUNV NP and/or Z proteins [105,110,111]. One of the JUNV NP interactors singled out in these proteomic studies in human cells was DDX3 protein [105,111]. DDX3 is a DEAD (Asp–Glu–Ala–Asp) box RNA helicase that belongs to the DExD/H family of proteins, and it harbors both ATPase and RNA helicase activities. DDX3 is involved in multiple steps of RNA metabolism, including RNA transcription and initiation of translation in host cells [112], and facilitates replication or translation of different RNA viruses such as hepatitis C virus (HCV), among others [113,114,115,116,117]. In reference to JUNV, there is evidence that demonstrates that DDX3 positively impacts JUNV viral growth in cell culture. Experiments conducted in DDX3 knockout cells showed a significant reduction in virus yields. Thereafter, lentiviral-mediated reconstitution of DDX3 expression resulted in a significant recovery of JUNV infection rate, demonstrating the critical role of DDX3 in viral growth [111]. Interestingly, these results were observed in human cells infected with either the pathogenic Romero or the attenuated Candid#1 JUNV strains, suggesting a common mechanism regardless of the pathogenicity of the JUNV strain [111]. Nevertheless, the molecular determinants beyond the proviral role of DDX3 during JUNV infection are still unclear. Of note, several experiments have intended to explain the mechanism by which DDX3 would co-adjuvate arenavirus propagation. For example, by means of a TCRV cell-based translation assay, it was observed that translation of a synthetic TCRV mRNA analog was unaffected in DDX3-deficient cells, indicating no critical engagement of DDX3 in TCRV mRNA translation initiation [111]. On the contrary, LCMV minireplicon assay-based experiments demonstrated that the pro-arenaviral activity of DDX3 strongly depends on DDX3’s ability to promote viral RNA synthesis and that both DDX3 ATPase and helicase RNA-unwinding activities are involved in this function [111]. Therefore, it is possible that DDX3 might facilitate JUNV replication in a similar mode as it works for LCMV; however, further research is necessary to fully understand DDX3 role in JUNV infection.

### 3.3. Host Kinases Impacting on JUNV RNA Synthesis

Many viruses modulate cell survival networks as a strategy to avoid cell death and sustain viral replication. Particularly, the extracellular-signal-regulated kinases 1 and 2 (ERK1/2) have been implicated in viral replication and the regulation of the innate immune response against viral infections [118,119,120,121]. ERK1/2 belong to the family of mitogen-activated protein kinases (MAPKs) and integrate the Raf/MEK/ERK cascade, which is involved in gene expression and cell metabolism [122,123]. Initial reports have demonstrated that JUNV infection induces activation of ERK1/2 [124]. Moreover, treatment with the MEK inhibitor U0126 provoked a reduction in the expression of both the viral NP and the envelope GP as well as a strong decrease in the production of JUNV infectious particles in cell culture. Not only was ERK1/2 phosphorylated in JUNV but also in TCRV and Pichinde virus (PICV)-infected cells [124]. Thereafter, U0126 inhibitor also impaired the functional activity of an S segment analog in a TCRV minireplicon system, further supporting that MAPKs display an important role in arenavirus viral replication and/or transcription through a yet undefined mechanism [125]. Overall, these observations indicate that ERK1/2 activation is required for JUNV multiplication and suggest it is important for efficient viral RNA synthesis, although it still remains unresolved whether ERKs alternatively induce changes in the phosphorylation state of viral or cellular targets.

The phosphatidylinositol 3-kinase (PI3K)/Akt cascade is another signaling pathway hijacked by mammarenaviruses. PI3K phosphorylates Akt and participates in many cellular processes, including cell survival and differentiation, as well as it has been described to contribute to replication of several RNA viruses [126]. PI3K/Akt inhibition was reported to affect LCMV budding, and to a lesser extent, LCMV RNA synthesis [127]. In the case of JUNV, the PI3K/Akt signaling pathway was found to be activated at an early stage of infection. Blockade of the PI3K/Akt pathway resulted in a decreased viral growth and reduction of virus adsorption to cells, possibly due to the blockage on the recycling of transferrin cell-receptor at the plasma membrane level [128]. These data collectively illustrate the diverse roles of the PI3K/Akt pathway in different stages of both the OW and NW arenavirus life cycle.

### 3.4. JUNV Z Protein-Binding Partners Related to the Cellular Energy Metabolism Support Virus Propagation

A recent large-scale proteomics approach identified a series of human factors that associate to the JUNV Z protein, some of which were found in JUNV particles [110]. Bioinformatic analysis of the Z-binding partners exhibited a strong enrichment of several functional classes of human proteins including (i) ribosomal proteins, (ii) Rab family proteins and (iii) host factors involved in energetic metabolism pathways such as ATP or Purine biosynthesis. For example, inosine-5′-monophosphate dehydrogenase 2 (IMPDH2), which catalyzes the conversion of inosine 5′-phosphate (IMP) to xanthosine 5′-phosphate (XMP), a rate-limiting step in the de novo synthesis of guanine nucleotides [129], was required for optimal JUNV propagation as well as for JUNV VLP release, suggesting the involvement of IMPDH2 in viral assembly and/or budding [110]. Consistently, merimepodib (MMPD, VX-497), a potent inhibitor of IMPDH, had an antiviral activity against JUNV, as evidenced by a reduction in viral production [130]. Thereafter, experiments using a JUNV minireplicon assay further showed that a noncompetitive inhibitor of IMPDH reduced viral RNA synthesis and that siRNA-mediated knockdown of human IMPDH2 markedly affected minigenome-containing VLP production, thus suggesting a key role of IMPDH2 in viral RNA synthesis [131].

Another Z target, ATP6V0D1, a member of the multi-subunit vacuolar V-ATPases complex, was reported to be necessary to sustain JUNV growth. The fact that ATP6V0D1 was not found in JUNV VLPs and virions [110] may be suggestive that this ATPase operates early upon infection, probably cooperating in viral RNA synthesis. As previously mentioned, blocking the V-ATPase complex with bafilomycin A1 reduces JUNV multiplication [72]; therefore, it is feasible that JUNV could recruit ATP6V0D1 as a means to support viral RNA synthesis. In sum, all these findings are indicative that interaction of Z protein with certain host factors related to the cellular energy metabolism, such as IMPDH2 or ATP6V0D1, may positively impact on more than one step of the JUNV cycle.

### 3.5. Host Heterogeneous Nuclear Ribonucleoproteins (HnRNPs) Are Sequestered by JUNV NP and Are Required for Viral Growth

HnRNPs constitute a family of cellular RNA-binding proteins involved in processing pre-mRNA as well as in mRNA translation, trafficking and stability [132,133]. Several studies have ascribed relevant functions to hnRNPs in diverse viral infections including influenza and human immunodeficiency virus (HIV), among others [134,135,136]. Some hnRNPs have recently been identified as JUNV (and other arenaviruses) NP-binding partners [105,108,137,138]. Initial reports showed that hnRNP A1 associated to JUNV NP and that siRNA silencing of hnRNP A1 and A2 caused a decrease in JUNV protein synthesis and progeny virus production during acute infection [137]. Interestingly, it was also observed that intracellular localization of different hnRNPs, as well as their expression levels, could be altered upon viral entry to the cell. In the case of persistent JUNV infections, a marked reduction in the hnRNP A/B expression levels was observed, but there were no alterations in the nucleo-cytoplasmic transport of these hnRNPs. In contrast, in acute JUNV infections, hnRNP A/B expression levels were unaffected. However, hnRNP A1 relocated to the cytoplasm, whereas hnRNP A2 remained mainly in the nucleus [137]. hnRNP K was also detected as another JUNV NP interactor [105], validating previous siRNA knockdown experiments which demonstrated that hnRNP K is a necessary host factor required for JUNV multiplication [138]. Although hnRNP K total expression levels were unaffected upon viral infection, JUNV acute infection induced an increase in hnRNP K cytoplasmic localization, in contrast to JUNV persistently infected cells, where hnRNP K predominantly localized in the nucleus. Overall, the association of different hnRNPs to NP, together with their relocation to the cytoplasm and alteration of their expression levels, ultimately impact on viral growth. However, further studies such as replicon-based experiments would help to depict if the role of hnRNPs in the viral life cycle is specific to the RNA synthesis step.

### 3.6. Host Factors Involved in Regulation of JUNV Translation

The key regulatory step modulating RNA translation is the initiation stage, during which a number of eukaryotic initiation factors contribute to assemble a complete 80S ribosome at the start codon of the mRNA [139]. EIF4E is the component responsible for the recognition of the cap structure that is coupled to the mRNA. EIF4E associates with the scaffolding protein eIF4G and the eIF4A RNA helicase, jointly forming the eIF4F complex that is required for the onset of cap-dependent translation. Thereafter, the 60S ribosomal subunit is recruited to further assemble an 80S complete ribosome onto 5′ capped eukaryotic mRNA and fulfill translation.

In this regard, viruses have developed various strategies to take control of the host translation machinery at the initiation, elongation or termination steps [140,141,142]. Some use alternative noncanonical translation mechanisms by replacing cellular eIFs with viral proteins [143,144]. However, despite blocking eIFs, many RNA viruses still continue to produce capped mRNAs [145], probably to impart mRNA stability rather than to affect initiation of translation. In this sense, for several viruses, a balanced blend of canonical and noncanonical mechanisms seem necessary for a flawless translation process.

In the case of mammarenaviruses, viral mRNAs bear a capped 5′ untranslated region (UTR) and lack a 3′ poly(A) tail [39]. Therefore, it would be conceivable that JUNV exploits a conventional cap-dependent translation mechanism employing the eIF4E for the initiation of translation. However, evidence demonstrates that JUNV multiplication takes place independently of functional eIF4E, but it strictly requires the presence of both eIF4GI and eIF4A, as silencing of any of both factors caused a marked detrimental effect on viral protein synthesis and viral growth [146]. In contrast, silencing of eIF4E factor neither reduced viral NP synthesis nor virus yield in JUNV-infected cells, as similarly observed for TCRV [146]. In accordance, JUNV NP was found to colocalize in the cytoplasm with eIF4A and eIF4GI, but not with eIF4E [146]. Furthermore, results from a cell-based translation assay using synthetic TCRV mRNA analogs confirmed that viral translation depends on eIF4G, but displays a low dependence on the cap-binding initiation factor eIF4E [147]. Based on these observations, it is tempting to speculate that JUNV may engage alternative host factors for the onset of a noncanonical cap-dependent mechanism. Besides, mammarenavirus Z protein was reported to repress the translation of capped mRNAs by directly binding to eIF4E, suggesting that Z might act as a negative regulator of host mRNA translation initiation [148,149,150]. This resembles a strategy employed by several viruses which either block or substitute different eIFs to take control of the host translation machinery [141,143,144].

### 3.7. JUNV Hijacks the Autophagy Pathway and Exploits Lipid-Droplets to Successfully Accomplish Viral Infection

The autophagy process is triggered by different stress signals to restore cellular homeostasis and eliminate senescent material [151]. It has also been reported to contribute to the immune response against intracellular pathogens [152]. Autophagy induces dynamic membrane rearrangements within the cell, starting with the nucleation of the phagophore, which engulfs and sequesters cytosolic components to generate an autophagosome, the hallmark organelle of the autophagy pathway [153]. Thereafter, this newly-formed vesicle fusions with lysosomes forming mature autolysosomes, leading to digestion of the internalized material.

Viruses may take advantage of this architecture reorganization to promote viral replication and assembly, as already documented for several RNA viruses [154,155,156,157,158,159,160]. In the case of the OW Mopeia virus (MOPV) and LASV, autophagy has been suggested to play a positive role in the release of infectious particles [161]. As for JUNV, recent reports have determined the participation of the autophagy process in viral propagation. For instance, there is evidence demonstrating an increment of the LC3-II/LC3-I ratio in JUNV infected cells, as well as a degradation of the p62 autophagic receptor, suggesting a full completion of the autophagic flux [162,163,164]. Moreover, JUNV infection showed to induce the colocalization of p62, ATG16, RAB5, RAB7A and LAMP1 factors with LC3 protein, thus reinforcing the notion that phagosomes undergo the whole maturation process during viral infection [163]. Of note, the critical autophagy-related proteins ATG5 and ATG7, involved in the extension of the phagophoric membrane [165], and the pivotal autophagy regulator Beclin-1 [166] were found to be necessary for JUNV propagation, strongly pointing to a pro-viral role of the canonical autophagy pathway [162,163]. Collectively, these data demonstrate that JUNV promotes autophagy to establish infection; nevertheless, extensive research is needed to dissect the underlying mechanistic details beyond this deeply unexplored host-cell interaction mechanism.

Lipid droplets (LDs) are considered central hubs of the lipid homeostasis. They are highly dynamic monolayer spheres with the capacity to constantly change their size and location throughout the cell. LDs have been involved in RNA virus infections and in the host immune response [167]. For example, the capsid protein of several RNA viruses, such as Dengue, Zika or HCV associate with LDs, indicating their potential role as scaffolds for nucleocapsid formation during the encapsidation process [168,169,170]. Besides, it is also known that several viruses trigger the autophagic degradation of LDs, a process commonly known as “lipophagy”, as a way to release lipids and thereby liberate the energy required for virus replication [171,172]. Likewise, SARS-CoV-2 proteins and RNA were found associated with LDs in infected cells, suggesting that LDs are recruited to viral factories to fuel replication [167,173]. In line with these findings, recent evidence demonstrated a decline in the number of LDs during JUNV cell infection and revealed that compound-mediated blockade of LDs genesis significantly decreases extracellular virus production, confirming the relevance of these lipid subcellular structures to sustain JUNV growth [174]. These observations are compatible with earlier studies showing that treatments affecting the cellular general lipid metabolism restrain JUNV yields [175]. Altogether, these data highlight a possible interplay between LD catabolism and autophagy during JUNV infection, a research field that remains largely unexplored.

### 3.8. Traffic of Viral Components within the Cell: Virus–Host Teamwork in the Assembly of the Glycoprotein Complex into Mature Virions

During the vesicle transport within the cell secretory pathway, cargos accumulate in specific membrane sites of the donor compartment and are incorporated into delivery vesicles covered with either coatamer protein I (COPI), COPII or clathrin [176,177]. These three classes of carriers mediate alternative stages of the secretory circuit. In regard to the intracellular transport of JUNV components, several vesicular trafficking proteins were detected as Z-binding partners, either in Z VLPs and/or in JUNV particles [110]. For example, the ADP-ribosylation factor 1 (ARF-1), a GTP-binding protein involved in transport within the ER–Golgi complex [178], was found in JUNV particles and proved to be necessary for JUNV multiplication, suggesting it intervenes in the delivery of Z (or additional viral proteins) to sites of virus assembly [110].

The arenavirus glycoprotein complex is translated in the ER as a single precursor GPC and it undergoes *N*-linked glycosylation. Cleavage by host Subtilisin Kexin Isozyme 1/Site 1 Protease (SKI-1/S1P) in the trans-Golgi apparatus renders GP1, GP2 and SSP subunits, which assemble into trimers, constituting the spikes on the virion envelope [37,179]. Recent evidence demonstrated that the impaired processing of GPC can activate its aggregation in the ER through the formation of inter-chain disulfide bonds [180]. Interestingly, variations in the glycoprotein processing were observed among different JUNV strains, as attenuated Candid#1 GPC showed to be less efficient than pathogenic Romero GPC in progressing through the ER folding checkpoints, prior to being exported to the Golgi apparatus. Noteworthy, a single amino acid substitution (T168A) within an *N*-linked glycosylation motif of GP1 in Candid#1 with respect to Romero, was found to be responsible for GPC retention in the ER [180]. Moreover, augmented levels of the LC3II/total LC3 autophagy marker were detected in Candid#1 infected cells, suggesting that aggregated proteins sequestered in the ER could be cleared in the cytoplasm by an autophagy-dependent lysosomal pathway [180].

Additionally, the interplay between JUNV GP and the ER–Golgi Intermediate Compartment (ERGIC) 53 kDa protein (ERGIC-53) has been documented [181]. ERGIC-53 functions as a cargo receptor for glycoprotein trafficking within the early exocytic pathway. It interacts with the GPC precursor, but not with the proteolytically processed GP1 and GP2 subunits, indicating that the interaction takes place in the ER or ERGIC, prior to GPC proteolysis by SKI-1/SP1 in the Golgi apparatus. ERGIC-53 relocates to the plasma membrane during JUNV infection, is incorporated into JUNV particles and is crucial to sustain viral infectivity [181]. Indeed, loss of ERGIC-53 does not impair viral morphogenesis, but reduces infectivity, which might be linked to the proper maturation of JUNV GP or failure in traffic of cellular factors required for virion structure [181].

It is important to mention that the adequate incorporation of GP into virions also seems to be dependent on the lipid-profile at the plasma membrane, as experiments applying cholesterol depletion showed a drop in JUNV GP association with the cell membrane, together with a reduction in virus yields, suggesting that JUNV GP might need to associate to cholesterol enriched membrane microdomains -also known as lipid rafts- for a competent assembly of infectious particles [182]. On the contrary, other studies employing electron microscopy reported that Candid#1 GP clusters into discrete microdomains which are different from lipid-rafts [183]. Besides these findings, by applying a high-resolution flow virometry assay, significant differences in JUNV particle infectivity were reported according to the lipid membrane composition of the infected cells [184]. Altogether, these data highlight the importance of the protein and lipid-pattern at the plasma membrane level and the need of further studies to fully characterize the impact of membrane microdomains composition in JUNV infectivity.

### 3.9. Cellular Targets Required for an Efficient Release of JUNV Particles

**Figure 2:**
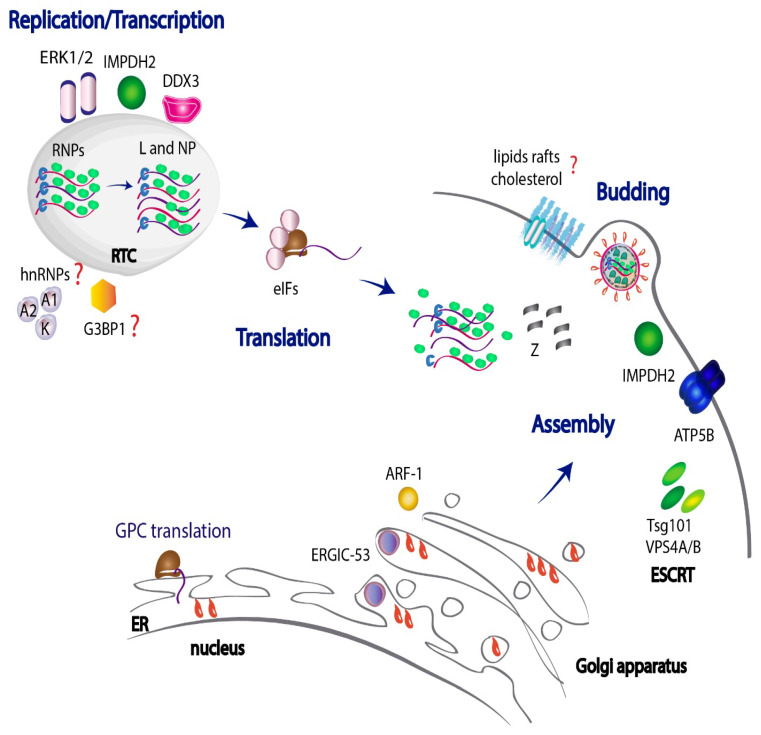
Host factors contributing to JUNV replication, assembly and budding processes. The JUNV genome is enwrapped by multiple copies of the viral nucleoprotein (NP), forming viral ribonucleoprotein complexes (vRNP), also known as nucleocapsids, which tightly associate to the viral L RNA-dependent RNA polymerase. They constitute the biologically active units for transcription of subgenomic viral messenger RNA (mRNA) as well as for viral genome replication. Both events take place in discrete puncta structures within the cytoplasm called replication–transcription complexes (RTCs). Several host factors, such as extracellular-signal-regulated kinases 1 and 2 (ERK1/2), inosine-5′-monophosphate dehydrogenase 2 (IMPDH2) and DEAD-box helicase 3 (DDX3) were described to contribute to JUNV RNA synthesis. The stress granule (SG)-associated Ras GTPase-activating protein-binding protein 1 (G3BP1), a phosphorylation-dependent endoribonuclease that modulates RNA metabolism, concentrates in cytoplasmic punctuated structures and colocalizes with NP, presumably being recruited to RTCs. Other targets, such as heterogeneous nuclear ribonucleoproteins (hnRNPs), relocate to the cytoplasm, associate to NP and facilitate viral propagation. However, it is still not clear whether they specifically interfere in the viral replication step. JUNV translation also takes place in the cytoplasm and critically depends on eukaryotic initiation factor 4G (eIF4G) but displays a low dependence on the cap-binding initiation factor eIF4E. Glycoprotein precursor (GPC) intracellular transport through the ER–Golgi pathway is aided by host factors ADP-ribosylation factor 1 (ARF-1), a GTP-binding protein involved in trafficking within the ER–Golgi complex, and ER–Golgi Intermediate Compartment 53 kDa protein (ERGIC-53), which functions as a cargo receptor for delivery of the GPC to budding sites. The adequate incorporation of GP into virions also depends on the lipid profile at the plasma membrane, affected by variations in the content of cholesterol and lipid-rafts microdomains. The viral Z matrix protein is the main force driving virus budding and release. This protein hijacks several host targets, such as IMPDH2 and the ATP-synthase ATP5B, to facilitate viral egress and complete the viral cycle. Components of the host endosomal sorting complex required for transport (ESCRT), including tumor susceptibility gene 101 protein (Tsg-101) and vacuolar protein sorting 4A or 4B (VPS4A/B), are also strictly required to sustain JUNV budding.

There is evidence that several host targets are hijacked by JUNV to facilitate viral egress and complete the viral cycle. In addition to IMPDH2, Z-binding partners impacting on JUNV release also include ATP5B, an ATP synthase that localizes to the plasma membrane. ATP5B is necessary to sustain virus budding, as shown by ATP5B depletion experiments, which resulted in a significant reduction in JUNV VLP egress [110]. In addition to this, Tsg101 protein, a component of the Endosomal Sorting Complex Required for Transport (ESCRT), has also been implicated in JUNV and other arenaviruses budding [17,95,185]. The ESCRT machinery is a protein network that helps sort endosomal cargo in vesicles budding into the lumen of multivesicular bodies (MVB) [186,187]. Tsg101 participation in JUNV release is likely to be mediated through its interaction with Z protein PTAP (Pro–Thr–Ala–Pro) late motif located at the C-terminal domain. This motif was shown to be crucial for efficient arenavirus budding [93,97] as also similarly reported for other RNA viruses [188]. Evidence demonstrated that Tsg101 protein is present in JUNV virions and that the compound-mediated blockade of JUNV-Tsg101 interaction impairs JUNV VLP egress [17]. Further experiments depleting the ESCRT accessory proteins Vacuolar Protein Sorting 4A (VPS4A) or VPS4B, which are involved in the late steps of the endosomal MVB pathway, also abrogated the efficient release of infectious JUNV particles, once again confirming that the ESCRT complex is required for an effective JUNV budding process [110] (Figure 2).

## 4. Host Immune Response upon JUNV Infection

AHF is characterized by severe hemorrhagic manifestations, together with a robust IFN-I response and the production of high levels of proinflammatory cytokines, such as IL-6, IL-8 and TNF-α, as well as the anti-inflammatory IL-10. The severity of the disease has been correlated with elevated serum levels of IFN-α and cytokines [189,190,191]. In contrast, the HF caused by the OW mammarenavirus LASV is characterized by a strong decrease in levels of IFN-I and proinflammatory cytokines [192]. IFN-I is the first line of defense against viral infections. It is a key component of the innate immune system, and it includes several subtypes, namely, IFN-α and IFN-β, among others [193]. The IFN-I response is triggered by pathogen-associated molecular patterns (PAMPs), unique molecular features generated upon infections, which are recognized by the immune system through host pattern recognition receptors (PRRs). PRRs can either be cytosolic, as retinoic acid-inducible gene I (RIG-I)-like receptors (RLRs), or nucleotide oligomerization domain (NOD)-like receptors, or associated to membranes, as Toll-like receptors (TLRs). PRRs activate downstream signaling that ultimately leads to an IFN-I-stimulated antiviral state [194]. As for JUNV, viral RNAs may fold into structures that could potentially be recognized as PAMPs, such as double-stranded RNA (dsRNA) or stem-loops at the intergenic regions. In this regard, it has been demonstrated that JUNV is sensed by different host PRRs, including the cytosolic RIG-I, the melanoma differentiation-associated protein 5 (MDA5) and the protein kinase R (PKR) RLRs, as well as by the transmembrane TLR2/TLR6 heterocomplex.

### 4.1. RIG-I and MDA-5 Sense JUNV and Activate the IFN-I Cascade

Upon exposure to a dsRNA ligand, RIG-I and MDA-5 receptors undergo conformational changes and bind to the mitochondrial antiviral signaling protein (MAVS). Activation of MAVS results, in turn, in the activation and translocation of the interferon responsive transcription factors (IRF)-3, IRF-7 and/or nuclear factor kappa B (NF-κB) to the nucleus of the infected cells. This results in IFN-I production [193,194]. Cumulative evidence demonstrated an association of JUNV infections with this signaling pathway. For example, infection of human lung epithelial cells with the vaccine Candid#1 strain induced IFN-I production, which was shown to be dependent on RIG-I and MDA-5 upregulation and associated with moderate levels of IRF-3 nuclear translocation [195]. Moreover, both RIG-I and MDA-5 colocalized with dsRNA and NP in distinct punctate structures [196], suggesting JUNV’s NP direct interaction with these PRRs, as previously reported for LCMV arenavirus [197]. IFN-β signaling upon JUNV infection was also shown to be dependent on MAVS, as IFN-β levels were abrogated in Candid#1-infected MAVS-knockout cells, resulting in higher viral titers [198]. Similarly, JUNV pathogenic strains also triggered IFN-I release and RIG-I upregulation in human infected cells [195,199,200].

### 4.2. Pathogenic and Nonpathogenic JUNV Strains Activate PKR Differently

PKR is a PRR ubiquitously expressed at basal levels in the cytoplasm. Upon viral infection, PKR recognizes virus-derived dsRNA and undergoes activation through dimerization and autophosphorylation. Phosphorylated PKR (phospho-PKR), subsequently phosphorylates the translation initiation factor 2 (eIF2), leading to global host translation shutdown. In addition, PKR modulates the antiviral IFN-I response [201]. During JUNV infection, PKR senses dsRNA and becomes activated. This is evidenced by an increase in total amounts of PKR and its phosphorylated form, as well as by the redistribution of PKR to NP-containing punctuated structures that also colocalize with dsRNA [105,196,198,202,203]. These observations pinpoint the hypothesis that PKR–NP interaction may be occurring within RTCs. Consistently, biochemical assays have demonstrated that PKR is a JUNV NP binding partner [105]. However, the relation of JUNV RTCs with the host immune system still needs to be elucidated.

Of note, infections with pathogenic or attenuated JUNV strains showed fundamental differences in the activation of the PKR pathway. For instance, the Romero strain caused a robust phosphorylation of PKR and eIF2 and a substantial host translational suppression [202]. Infected PKR-knockout cells showed slightly diminished viral titers and augmented expression levels of RIG-I and ISG-15 [202]. This suggested that pathogenic strains might activate PKR to attenuate the IFN-I response. Conversely, the Candid#1 strain was neither capable of inducing eIF2 phosphorylation nor protein synthesis shutdown despite the high levels of activated phospho-PKR [105,196,198]. Interestingly, in PKR-deficient cells, propagation of Candid#1 was not affected compared to wild-type cells [105,198]. The strong colocalization observed between Candid#1 NP, dsRNA and phospho-PKR, not reported for the Romero strain, might be indicative that NP of Candid#1 directly interacts and, hence, interferes with phospho-PKR functionality [105,196,203]. Further studies are required to depict the differences in the impact on protein synthesis among infections with pathogenic versus nonpathogenic JUNV strains.

Interestingly, infection with the nonpathogenic TCRV upregulated PKR (and consequently phospho-PKR), which also colocalized with NP, resembling JUNV strain behavior [198]. Unlike the Romero strain, TCRV progeny production significantly increased upon infection of PKR-deficient cells, along with reduced intracellular levels of IFN-I mRNA transcripts, revealing a role of PKR in controlling TCRV infection [198,204].

### 4.3. JUNV Is Sensed by TLR Membrane Receptors in Mouse Macrophages

It has been reported that the JUNV vaccine strain Candid#1 is recognized by the TLR2/TLR6 heterocomplex in mouse macrophages, triggering a series of signaling cascades that lead to the activation of NF-κB and the induction of IFN-β and TNF-α [52,205]. Consistently, TLR2 knockout mice cleared Candid#1 more slowly than wild-type mice [205]. GP resulted to be the viral molecule responsible for this TLR-2-dependent innate immune response. Moreover, VLPs pseudo-typed with GP from either the pathogenic strain Parodi or nonpathogenic Candid#1 elicited a similar cytokine response [52], showing that the amino acid changes that attenuated Candid#1 GP did not impact on TLR recognition. Whether this mechanism can be extrapolated to human cells remains to be determined.

### 4.4. Interplay between JUNV and Several Host ISGs

Once synthesized, IFN-I is secreted and recognized by the transmembrane IFN-α/β receptor (IFNAR), either in an autocrine or paracrine manner. This IFN-I signaling pathway leads to the activation of multiple IFN-stimulated genes (ISGs) which, through a wide range of functions, amplify the IFN cascade and establish the antiviral state needed to counteract viral growth.

JUNV, as many other viruses, activates the expression of several ISGs. First, both the Candid#1 and Romero strains induce high levels of ISG15 as well as the expression and phosphorylation of the transcription factor signal transducer and activator of transcription 1 (STAT-1) [195,199], a hallmark event of IFN signaling [206]. The higher ISG15 mRNA and STAT1 phosphorylation levels observed in Candid#1-infected cells with respect to Romero suggested a more robust IFN response elicited by the vaccine strain [195]. JUNV infection also stimulated the ISG viperin, which seems to act as a restriction factor, as viperin-silenced cells led to higher levels of JUNV mRNAs [174]. In the same lane, exogenous overexpression of viperin turned cell cultures less permissive to JUNV propagation and led to a significant drop in viral RNA levels. Of note, evidence indicates that viperin localizes to LDs, which is a mechanism for viral inhibition [207]. As previously mentioned, considering that LDs play an important role during JUNV viral growth, this points to another possible mechanism for viperin as a JUNV restriction factor.

In cell cultures infected with Candid#1, also observed were increased levels and relocalization of the bone marrow stromal cell antigen-2 (BST-2), also known as tetherin [208]. This membrane-associated ISG restricts enveloped viral release from host cells by physically tethering them onto the cell surface [209]. In VLP-release experiments, it was observed that BST-2 strongly restricted Z-mediated VLP production by clustering VLPs on the plasma membrane. The co-expression of NP partially counteracted this effect, perhaps as a result from sequestration of BST-2 by NP [208].

Altogether, these data illustrate the extensive interaction of JUNV with different ISGs and the multifaceted strategies developed by the virus to modulate ISGs activity, circumvent the IFN-I response and ensure its own replication and spreading.

### 4.5. JUNV NP and Z Proteins Modulate the IFN-Pathway

Viruses have widely developed strategies to impair, escape and counteract IFN-sensing, interacting with cell signaling networks to promote their successful replication. For instance, the influenza A virus nonstructural protein NS1 restricts IFN-I induction either by binding viral dsRNA and, consequently, preventing recognition by PRRs or by directly blocking RLR sensors [210,211,212]. Similarly, nonstructural ORF-9b protein of SARS-CoV-2 was shown to interact with MAVS, limiting the host IFN response [213,214]. In line with these findings, arenaviral NP has also been identified as an IFN-I antagonist affecting multiple steps of the IFN response. In vitro assays demonstrated that plasmid-driven overexpression of NP from NW mammarenaviruses, such as JUNV, MACV and PICV as well as from OW LASV or LCMV, inhibits IRF-3 [215] and NF-kB [216] nuclear translocation, resulting in reduced IFN-β promoter activity. Moreover, NP from several mammarenaviruses, including JUNV, interact with the IκB kinase (IKK)-related kinase IKKɛ, and it was demonstrated that its kinase activity is inhibited in LCMV-infected cells [217]. NP has also been demonstrated to suppress the potentiation of RIG-I function mediated by the dsRNA binding protein PACT and the activation of IFN-β production in vitro [218].

Interestingly, the ability of arenavirus NP to suppress IFN-I production was associated to a DEDDh (Asp–Glu–Asp–Asp) motif in the C-terminal domain, which is conserved in the DEDDh family of 3′-5′ exonucleases [216,217,219,220]. Several reports provided evidence that NP from LASV, TCRV, MOPV and PICV exhibit exonuclease (ExoN) activity in vitro [221,222,223,224,225,226]. Moreover, recombinant LASV, MOPV or PICV carrying mutations in the active site of ExoN domain elicited a higher IFN-I response in comparison to wild-type recombinant virus, supporting the role of ExoN active site in regulating IFN-I production [226,227,228]. As for JUNV, although NP was able to suppress IFN-I signaling upon overexpression in cell culture [215], whether this is due to ExoN activity is still to be demonstrated. The crystal structure of the C-terminal domain of JUNV NP revealed a DEDDh exoribonuclease structure; however, no ExoN activity was detected in vitro [229]. The dsRNA accumulation occurring in pathogenic or nonpathogenic JUNV infections, which is not observed in LASV-infected cells, supports the idea of an inactive ExoN domain [196,203]. The study of the JUNV NP C-terminal domain functionality in the context of a viral infection is compelling to better understand its contribution in modulating the host immune response.

Not only NP but also arenavirus Z has been implicated in antagonizing the IFN-I response [230,231,232]. JUNV Z protein directly binds to RIG-I [230,231] and MDA-5 [231] in transfected cells. The interaction of Z with these PRRs prevents their association with MAVS and blocks the activation of NF-Kb and IRF-3 [230,232]. This suppression mechanism of the innate immune response seems to be common for all human pathogenic mammarenaviruses [232]. Altogether, these data illustrate the extensive interaction of JUNV NP and Z with key components of the innate immune system and the multifaceted strategies developed by the virus to circumvent the IFN-I response and ensure its own replication and spreading.

## 5. Reverse Genetics Systems as a Tool to Study JUNV Biology and Virus–Host Interactions

The development of reverse genetics systems for JUNV, as well as for other mammarenaviruses, has strongly facilitated the identification of molecular determinants involved in the viral cycle, particularly in viral replication and pathogenesis. Consequently, it has become an excellent tool for the study of virus–host interactions.

### 5.1. Rescue of Recombinant JUNV and Its Use in the Study of Virus Pathogenicity and Vaccine Development

Recombinant pathogenic JUNV (XJ13 strain) and Candid#1 vaccine strain were successfully rescued from cloned cDNA using a T7 polymerase-driven cell-based system. It consists of two plasmids, each encoding full-length cDNA copies of the viral S and L antigenomic segments [233,234]. Efforts were later directed to the development of Pol I/II (polymerase I/II)-driven reverse genetics systems to achieve the rescue of rJUNV in human cell lines already approved by the FDA for vaccine development [235,236]. Not only Candid#1 but also the pathogenic Romero strain were successfully rescued from plasmid transfection, evidencing the same growth properties in vivo as the corresponding parental strains. Importantly, infection with rRomero caused 100% lethality in an AHF guinea pig model, whereas rCandid#1 infection was asymptomatic and provided protection against lethal challenge with Romero [235].

The use of recombinant chimeras or viruses containing specific mutations within their genomes have provided valuable information in regard to JUNV pathogenesis determinants. For example, a Romero chimera where Candid#1 GPC was substituted for Romero GPC (rRom/GPC-Can) displayed a completely attenuated phenotype in guinea pigs [237]. In a similar way, a single amino acid substitution in the G2 glycoprotein transmembrane domain (F427I) was identified as the major attenuation determinant in mice infected with Candid#1 [238,239]. This mutation also resulted a high attenuation determinant in guinea pigs, though insufficient to prevent virus dissemination [237]. Furthermore, recent reports using rMACV and a rMACV chimera bearing the Candid#1 GPC have demonstrated the critical role that GP *N*-linked glycans play in arenavirus pathogenicity [240]. Of note, evidence indicates that Candid#1 attenuation is not just limited to the viral glycoprotein. For instance, rRomero carrying a single mutation in the Z protein RING domain (V64G) derived from the Candid#1 sequence, showed reduced viral growth in vitro and an attenuated phenotype in a guinea pig model [241].

As an alternative strategy in the search of novel vaccine candidates to combat JUNV, the latest studies were oriented to the development of reverse genetic systems for the rescue of the closely related nonpathogenic TCRV (rTCRV) [242,243]. These approaches included the generation of chimeric TCRV variants expressing JUNV attenuated strain glycoproteins (rTCRV/GPXJcl3) or the incorporation of attenuation markers within the 5′ noncoding terminal sequence from the S segment (rTCRVsNCR) [243]. Altogether, these advances show that recombinant DNA technology can be widely used as a powerful tool for the rational design of a safe vaccine platform against JUNV, decreasing the probability of potential reversion to virulence or establishment of a persistent infection.

### 5.2. Alternative Systems to Evaluate JUNV Replication, Assembly and/or Budding Processes

Simultaneously, several minigenome (MG) (also known as minireplicon) reverse genetics systems have been designed for JUNV and have been widely used to understand the molecular mechanisms underlying the viral replication process [13,131,233,235,236]. These platforms specifically allow the modeling of viral RNA synthesis, in contrast to the previously described infectious recombinant JUNV, oriented to the analysis of the whole virus life cycle. Minireplicon systems rely on a plasmid encoding a genome analog that mimics the natural viral genome and harbors reporter genes in place of viral proteins’ ORFs, which allows to monitor viral RNA synthesis. When the viral L and NP proteins are provided in trans, the MG is transcribed and replicated, followed by the expression of the reporter genes carried by the MG. These assays enable the study of the role of viral and/or host proteins in the replication stage and they are particularly attractive for assessing the effects of siRNA targeting different viral mRNA or compounds capable of disrupting viral RNA replication [131].

Further approaches have developed reverse genetics systems for the recovery of JUNV transcription and replication competent virus-like particles, known as trVLPs or infectious VLPs [244]. In these systems, the inclusion of GP- and Z-expressing plasmids, enables the production of fully encapsidated MG-containing particles that can efficiently infect new cells, as long as these cells express NP and L cofactors. Thus, VLPs recapitulate the assembly and budding stages, apart from viral RNA synthesis [13,131]. Likewise, earlier reports have previously described the development of JUNV Z-driven infectious chimeric TCRV VLPs, expressing JUNV glycoprotein on the surface [93]. In addition to this, the fact that the sole expression of Z protein induces the release of VLPs in the absence of other viral components [95,96,185], allowed the discrete study of viral morphogenesis and/or egress, independently of viral entry and replication [93,97,110,181]. Of note, it has been recently tested that JUNV Z efficiently produces VLPs in a variety of cell lines, unlike TCRV, which showed limited cell-type restrictions for the budding process [245]. In sum, all these cell-based systems described above have strongly helped to dissect different aspects of the viral life cycle of JUNV and related arenaviruses and have provided insights of JUNV interaction with cell host factors.

## 6. Conclusions

AHF is a potentially lethal, endemic disease affecting the population of most agricultural areas in Argentina. Currently, treatment with immune plasma from convalescent patients is the antiviral therapy being used to combat AHF. Even though this strategy has been demonstrated to strongly reduce case-fatality rates, it is associated with a potential late neurological syndrome in 10% of treated patients. Moreover, it presents serious difficulties in the adequate maintenance and flawless accessibility to immune plasma stocks. Interestingly, the combination therapy of the broad-spectrum antiviral nucleoside analogs, ribavirin and favipiravir (T-705), has demonstrated clinical success in treating LASV, if administered early after infection. [246]. More importantly, a recent case report showed that this therapy was effective in treating JUNV infection, even when administered after 13 days of the onset of symptoms. [247]. This reinforces the need in the advancement of development and administration of novel antiviral strategies to battle JUNV, as an alternative to the conventional treatment.

A variety of studies on virus–host interactions have identified a series of cellular factors involved in JUNV infection. These findings have widely contributed to a better understanding of the virus–host interplay and the mechanisms underlying the different steps of JUNV life cycle spanning the entry, replication, assembly and budding processes. On the one hand, several reports have provided evidence that JUNV not only use the well-characterized hTfR1 receptor to enter the cell, but it also utilizes lectins, VGCCs and PS receptors as alternative entry factors. Probably, the use of these noncanonical receptors rises as a viral strategy to facilitate virus binding to cells, promote membrane fusion and enhance the viral cell tropism, therefore improving the overall efficiency of the entry process. Thereafter, clathrin-mediated endocytosis and subsequent transit through Rab5-early and Rab7-late endosomes stand as the sole convergent pathway opted by JUNV to fulfill entry to the cell (Figure 1).

In a similar way, many host candidates have emerged as viral-binding partners co-adjuvating JUNV replication. The DEAD-box helicase DDX3, the ERK1/2 kinases and the IMPDH2 dehydrogenase have raised as salient cellular targets contributing to JUNV viral RNA synthesis (Figure 2). Other targets such as hnRNP A1/A2 and hnRNP K and the ATP6V0D1 ATPase have also been well characterized as facilitators of viral propagation, however the fine-tuned mechanism beyond their participation in JUNV cycle still needs to be further clarified. Also, the ER–Golgi compartments are known to take part in JUNV assembly, as evidence demonstrated that the ER-associated ERGIC-53 cargo protein is necessary for the adequate incorporation of GP into JUNV mature virions. Likewise, ARF-1 factor, involved in trafficking different components along the ER–Golgi complex, is thought to intervene in the delivery of Z or additional viral proteins to different sites of virus assembly. In regard to the budding process, robust data support the knowledge that the ESCRT components Tsg101 and VPS4A/B display an important role in virus egress. In addition, other Z-binding partners localizing at the plasma membrane, such as the IMPDH2 dehydrogenase and the ATP5B synthase, were also shown to contribute to JUNV budding (Figure 2).

JUNV, as well as other mammarenaviruses, not only utilize the host cellular machinery to accomplish viral entry, replication and budding, but also manipulate host defenses to distract the immune response and drive successful multiplication and spreading. JUNV-induced IFN suppression may be regulated at multiple levels, varying with the viral strain, cell type and stage of infection. At the cellular level, the multifunctional viral NP stands as the main responsible factor modulating the antiviral state elicited upon virus infection. This protein has evolved to directly interact with several host partners and counteract the innate immune response. Z protein has also developed redundant and specific interactions to suppress IFN-I signaling, possibly by taking advantage of its versatile functionalities. The variations observed between pathogenic and nonpathogenic strains of JUNV may be due to the differences in the interactions of NP and Z proteins with the immune system. The deep knowledge of the diverse host–viral interactions operating for different JUNV strains will allow the development of specific antivirals and a better control of AHF disease.

Altogether, the information presented in this review highlights the importance of understanding the role of virus–host interactions in the successful completion of JUNV productive infection. There is a plethora of evidence demonstrating that this virus constantly demands support from the host to accomplish each step of its life cycle. To date, research efforts have mostly been oriented to the identification of host targets involved in the earlier steps of JUNV infection—particularly in viral entry and genome replication—as well as to the discovery of drug compounds inhibiting these processes. However, little progress has been made in regard to clinical trials in patients. Indeed, further detailed pre-clinical studies are required to fully comprehend the molecular mechanisms beyond some of the promising compounds with already demonstrated antiviral activity against JUNV and other pathogenic arenaviruses.

In addition to this: considering that JUNV is a rodent-borne virus, variations in certain environmental factors could allow JUNV to progressively expand its endemic area. For instance, the intrinsic properties of its natural host reservoir, with high reproductive-rates, together with dynamic modifications in farming practices or unpredictable ecological changes, could negatively impact on AHF incidence and distribution. In this regard, there is a constant risk of emergence of AHF disease in new zones, with the potential threat of new JUNV variants emerging, as regularly reported for pathogenic arenaviruses in America [248,249,250,251]. Therefore, it is compelling to orient research to the development and implementation of novel antiviral therapies to control JUNV and other mammarenavirus of public health concern.
